# Visual Function and Macular Carotenoid Changes in Eyes with Retinal Drusen—An Open Label Randomized Controlled Trial to Compare a Micronized Lipid-Based Carotenoid Liquid Supplementation and AREDS-2 Formula

**DOI:** 10.3390/nu12113271

**Published:** 2020-10-26

**Authors:** Pinakin Gunvant Davey, Thomas Henderson, Drake W. Lem, Rebecca Weis, Stephanie Amonoo-Monney, David W. Evans

**Affiliations:** 1 College of Optometry, Western University of Health Sciences, Pomona, CA 91766, USA; drake.lem@westernu.edu (D.W.L.); samonoomonney@westernu.edu (S.A.-M.); 2 Eye Clinic of Austin, Austin, TX 78731, USA; thendersonmd@outlook.com (T.H.); rweis@eyeclinicofaustin.com (R.W.); 3 VectorVision/Guardion Health Sciences, San Diego, CA 92128, USA; devans@vectorvision.com

**Keywords:** age-related macular degeneration, macular degeneration, macular pigment, MPOD, contrast sensitivity, medical food, carotenoids, lutein, zeaxanthin, meso-zeaxanthin, Lumega-Z, AREDS-2, PreserVision

## Abstract

Purpose: To compare the changes in visual and ocular parameters in individuals with retinal drusen who were treated with two commercially available nutritional supplements. Methods: An open-label, single-center, randomized, parallel-treatment with an observational control group design was utilized. The treatment groups included individuals with fine retinal drusen sub-clinical age-related macular degeneration (AMD), while the control group consisted of ocular normal individuals. The treatment groups were randomly assigned to the micronized lipid-based carotenoid supplement, Lumega-Z (LM), or the PreserVision Age-Related Eye Disease Study 2 (AREDS-2) soft gel (PV). Visual performance was evaluated using the techniques of visual acuity, dark adaptation recovery and contrast sensitivity, at baseline, three months, and six months. Additionally, the macular pigment optical density (MPOD) was measured. The control group was not assigned any carotenoid supplement. The right eye and left eye results were analyzed separately. Results: Seventy-nine participants were recruited for this study, of which 68 qualified and 56 participants had useable reliable data. Of the individuals who completed this study, 25 participants belonged to the LM group, 16 belonged to the PV group, and 15 to the control group. The LM group demonstrated statistically significant improvements in contrast sensitivity function (CSF) in both eyes at six months (*p* < 0.001). The LM group displayed a positive linear trend with treatment time in CSF (*p* < 0.001), with benefits visible after just three months of supplementation. Although there was a trend showing improvement in CSF in the PV group, the change was not significant after a Bonferroni-corrected *p*-value of *p* < 0.00625. Visual acuity, dark adaptation recovery and MPOD did not significantly improve in either treatment groups. Conclusion: The LM group demonstrated greater and faster benefits in visual performance as measured by CSF when compared to the PV group. This trial has been registered at clinicaltrials.gov (NCT03946085).

## 1. Introduction

The macular pigment is composed of three carotenoids: lutein, zeaxanthin, and meso-zeaxanthin [1,2]. They are responsible for the fovea’s yellow pigmentation and are densely concentrated within the axons of photoreceptors inner plexiform and outer plexiform layers at the center of the macula [1,2,3,4,5]. The two carotenoids, lutein and zeaxanthin, can only be acquired through dietary intake and cannot be synthesized within the body [2,6,7]; sources include vegetables, spinach, corn, and egg yolks [2,8]. Although some foods such as salmon skin, sardine skin, trout skin and trout flesh are known to have meso-zeaxanthin [9], the serum level of meso-zeaxanthin in healthy individuals is approximately 0.0003 µmol/L [10]. Unless individuals are artificially supplemented [10,11], meso-zeaxanthin in human eye is a byproduct of the conversion of lutein in retinal pigment epithelium [2,5,6,12,13,14]. Macular carotenoids constitute the macular pigment optical density (MPOD) and are associated with maintaining retinal health and optimal visual performance [2,3,8,15], suggesting the level of MPOD is an important biomarker in health and disease states. 

The MPOD’s protective capabilities have led researchers to investigate the role of carotenoids in the development of eye diseases, such as age-related macular degeneration (AMD) [16,17,18,19]. Prior reports have shown that oral supplementation of carotenoids can increase MPOD levels [20,21,22,23,24,25,26]; however, the duration of supplementation needed and the degree to which the increase in MPOD changes the visual performance have varied between studies, thus requiring further investigation to establish an optimal carotenoid supplement and delivery method for individuals with AMD.

The Age-Related Eye Disease Study 2 (AREDS-2) [27] evaluated the effects of a carotenoid multivitamin supplement and concluded that the AREDS-2 oral supplement proved to be efficacious in attenuating the progression of intermediate dry AMD to advanced AMD [27]. The AREDS-2 formula that is currently commercially available is PreserVision™, a soft-gel capsule (Bausch Health, Bridgewater, NJ, USA). 

One school of thought is that carotenoid vitamin therapies typically administered by conventional soft-gel formulas may be limited due to their efficacy of absorption (EOA) rates [28], prompting researchers to investigate more efficacious delivery systems with improved absorption [29,30]. Lumega-Z (Guardion Health Sciences Inc., San Diego, CA, USA) is a micronized lipid-based liquid carotenoid supplement that is commercially available. Micronization is a process which reduces the diameter of a solid material’s particles, which allows for easy absorption of nutrients to the blood stream. This process is the key factor in bioavailability, and ultimately, the effectiveness of the nutrients. Recently, the efficacy of Lumega-Z (LM) was compared to the AREDS-2 supplement PreserVision (PV; Bausch & Lomb, Rochester, NY, USA) in a group of healthy individuals [10]. The study reported changes in the serum carotenoid uptake, along with changes in contrast sensitivity function and MPOD as measured by heterochromatic flicker photometry [10]. They found that there was greater serum uptake and increase in MPOD in the group that used the LM supplement when compared to the PV [10]. There are no reports to date on the efficacy and clinical benefits of the LM supplement on individuals with retinal drusen who are at risk of AMD. 

The primary hypothesis tested was that the micronized lipid-based liquid carotenoid supplement would improve the visual function and MPOD greater than the soft-gel capsules of PV. The aim of this study was to clinically evaluate the benefits of two commercially available supplements in a head-to-head comparison in a group of individuals with retinal drusen and risk of AMD. 

## 2. Material and Methods

This study was approved by the institutional review board at Salus IRB, Austin, TX, USA ((www.salusirb.com; +1-512-380-1244) PI Thomas Henderson MD Protocol #1. Submission date 27 October 2017, approval date, 06 November 2017, amended protocol version #2, approval date 17 January 2018) and conducted in accordance with the principles of the Declaration of Helsinki. The complete date range for participant recruitment and follow up were between 19 January 2018 and 1 November 2018. The authors confirm that all ongoing and related trials for this intervention are registered. Accordingly, this trial has been registered at clinicaltrials.gov (NCT03946085). 

### 2.1. Participant Eligibility

All participants provided a signed consent and were recruited from a single clinic, under the care of an ophthalmologist at the Eye Clinic of Austin, TX. All participants underwent a comprehensive, dilated ocular examination to determine ocular health. The presence of retinal drusen was determined using ophthalmoscopic evaluation, and additionally recorded using a fundus camera Visucam PRO^TM^ (Carl Zeiss Meditec, Dublin, CA, USA).

Exclusion criteria were: (1) presence of diabetic retinopathy, macular edema, or other congenital retinal pathologies that may impact retinal measurements; (2) prior history of retinal detachment or vitreoretinal surgeries or drusen >63 micron in the central retina within 2 disc diameters of the fovea; (3) individuals within the immediate post-operative follow-up period (3 months) of cataract surgery or history of invasive ocular procedures such as anti-VEGF injections and (4) individuals with worse than 20/40 best-corrected visual acuity.

All measurements mentioned below were performed during each study visit, that is at baseline and three-month and six-month treatment duration. Manufacturers’ guidelines for reliability were utilized for each measure and when clinical measures were found not reliable, subjects’ clinical data were not included for the study analysis. 

### 2.2. Subjects and Supplementation in Different Groups

**Figure 1:**
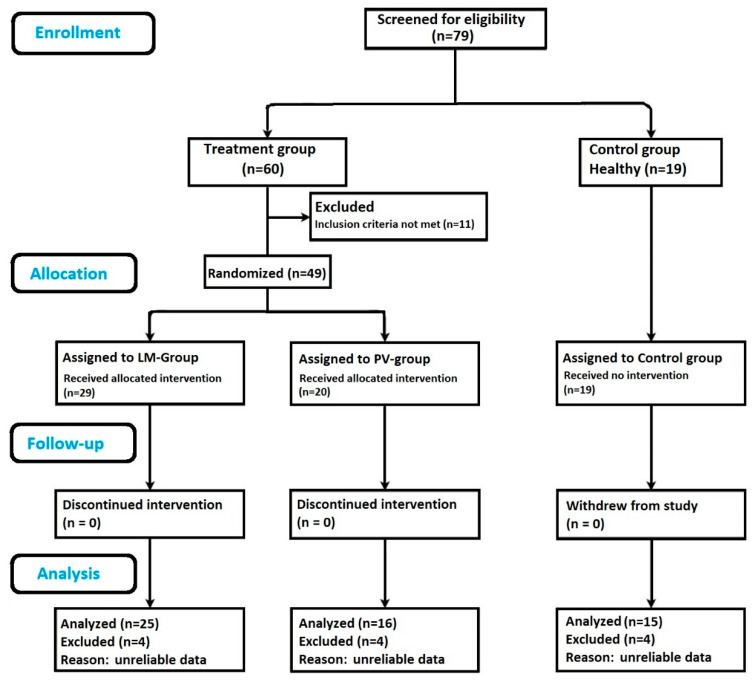
Flow chart showing study outline: subject enrollment, randomization, follow up and sample size analyzed. Lumega-Z (LM), PreserVision (PV).

The study participants included 79 adults in total: 60 participants with retinal drusen, and 19 ocular normal controls. Of the participants with retinal drusen, 11 individuals did not meet the visual acuity criteria and were excluded from this study. The treatment group was randomized approximately in a 1.5:1 ratio with the LM group (*n* = 29), or the PV group (*n* = 20). Since the efficacy of the LM supplement was not yet established in individuals at risk for AMD, the allocation ratio was chosen to be greater in the LM group. The flow chart in Figure 1 delineates participant assignments within each group. Four participants in each group did not have reliable data as per the manufacturer’s guidelines and were removed from this study. This brought the final numbers to: LM group, *n* = 25; PV group, *n* = 16; and control group, *n* = 15.

Study participants were instructed to not change their diets during the course of study. Both the treatment groups were provided their respective oral supplements at no cost for six months, to be taken daily with food. Appendix A provides the complete list of nutrients in the LM and PV supplements. The LM supplement (Guardion Health Sciences Inc., San Diego, CA, USA) contains lutein (15 mg), zeaxanthin (3 mg), and meso-zeaxanthin (10 mg) [10,31] taken orally in liquid form with two omega-3 fatty acids capsules (EPA and DHA 905 mg) daily [10,31]. The omega-3 fatty acid capsules contained fish oil concentrate—1260 mg of Alaska Walleye Pollock (*theragra chalcogramma*). The omega-3 fatty acid capsules used in this study were of ethyl ester type. Additionally, the LM supplement has other micronutrients (see Appendix A). The PV supplement (Bausch & Lomb, Rochester, NY, USA) is based on the AREDS-2 study formula and contains two carotenoids (10 mg lutein, 2 mg zeaxanthin) along with vitamin C, vitamin E, zinc and copper [10,27]. 

### 2.3. Visual Acuity Measurements

Visual Acuity was measured using the Early Treatment Diabetic Retinopathy Study (ETDRS) chart-based acuity test (Good-Lite Co., Elgin, IL, USA). Participants were tested at a distance of 4 m and instructed to read sequences of letters within a specified row, one eye at a time. The ETDRS test details were described previously [32] and the chart luminance was calibrated to 85 cd/m^2^, as recommended for photopic conditions [32,33]. The LogMAR measurements for each eye were obtained in accordance with ETDRS chart scoring protocols and visual acuity measures recorded [32].

### 2.4. Contrast Sensitivity Measurements

The VectorVision CSV-1000E device (Greenville, OH, USA) was used to measure contrast sensitivity function (CSF). Measurements were obtained via standardized patient procedures described in prior publications [33,34,35]. Briefly, contrast sensitivity is measured with participants using their best correction and viewing the chart at 2.5 m distance. The chart is rear illuminated (mean luminance 85 cd/m^2^) and presents a series of achromatic sine-wave targets of varying spatial frequency, incorporating 3, 6, 12, and 18 cycles per degree (CPD) in four separate rows [33,34,35]. Across each row, vertical target pairs with eight different contrast levels, respectively, are present and scores are recorded in log contrast sensitivity [33,34,35].

The participants were presented a suprathreshold example of a test pattern, and subsequently instructed to identify the test pattern between two targets presented on different rows [33,34,35]. This was continued to the next level of contrast threshold and spatial frequency. The results were scored according to the last column sine-wave target, correctly identified for each spatial frequency.

### 2.5. Dark Adaptation Recovery Measurements

Dark adaptation recovery (DAR) measurements were obtained using the MacuLogix AdaptDx adaptometer (MacuLogix Middletown, PA, USA). Details about the testing procedure and device methodology have been previously described in detail elsewhere [36,37,38,39,40]. Briefly, measurements were performed in a darkened room with the chin stabilized on a rest, fully dilated pupils and under monocular viewing conditions [36,37,38,39,40]. Testing begins with photo-bleaching using a wavelength of 505 nm to pre-condition the retina into a photobleached state and subjects were instructed to fixate on a target light (635 nm). Subsequently, a stimulus wavelength of 505 nm was presented at a fixed location and, using a 3-down/1-up modified staircase estimate design, and threshold was determined. Subjects were instructed to respond via a handheld response button when the stimulus was visible [37,38,39,40,41]. Upon completion, threshold values were reported as patient visual sensitivity units (dB) as a function of time, subsequent to the initial exposure, defined as the Rod Intercept Time [36,37,38,39,40]. The procedure was repeated in the fellow eye when applicable. 

### 2.6. Macular Pigment Optical Density Measurements

The MPOD was measured using the MapCatSF (Guardion Health Sciences, San Diego, CA, USA). The device uses heterochromatic flicker photometry to determine the level of blue light absorption in the macular pigment, described in detail elsewhere [41]. During testing, two wavelengths—455 nm (close to peak light absorption by macular pigment) and 515 nm (very low absorption)—are alternated within a circular 1.5° diameter stimulus, which is generally reported as flickering by the subject [41]. The null point is determined by adjusting the intensity of the 455 nm source until the subject reports no flickering. The 1.5° stimulus is then replaced with a 15° diameter stimulus, and the subject adjusts the intensity of the 455 nm source until no flicker is perceived around the periphery of the stimulus (some residual flicker will still be present in the center of the stimulus.) From the two intensity settings, central and peripheral, a microprocessor calculates the MPOD at the peak wavelength, 460 nm. This essentially represents the average value within the central 1.5°. The procedure is repeated in the fellow eye when applicable.

### 2.7. Optical Coherence Tomography

All participants had undergone testing with Zeiss Cirrus spectral domain optical coherence tomography (OCT; Carl Zeiss Meditec, Dublin, CA, USA). The OCT protocol macular cube 512 × 128 that scans the central 6 mm × 6 mm in an eye of axial length 24.4 mm was utilized. All OCT scans had a minimum quality score of 6 and were analyzed by a single investigator (PGD). The OCT’s standard software (SW Ver: 8.1.0.117, Carl Zeiss Meditec, Dublin, CA, USA) was utilized for analysis. The macular cube protocol automatically places an Early Treatment of Diabetic Retinopathy Study (ETDRS) grid centered on the fovea with a total of 9 sectors (1 foveal, 4 inner macula and 4 outer macula). The thickness between the internal limiting membrane and the retinal pigment epithelium is recorded in microns and is automatically calculated. Additionally, the thickness values in all sectors are compared to the reference database and sectors are assigned a percentile rank in the normal distribution. The output color codes each sector of the ETDRS grid and identifies it as follows: >99%, >95 but less than 99%, 95%, <5 but greater than1% or <1% of diversified distribution of normal population. The sectors that were not within the 95% of normal distribution were considered to be “abnormal” as they have a lower likelihood of being normal. 

### 2.8. Outcome Measures

The primary outcome measures were visual function as determined by visual acuity, contrast sensitivity and dark adaptation. Changes in visual performance were measured at three-month and six-month duration after supplement intake and compared with the baseline measurements. Additionally, the MPOD was evaluated at the baseline, three- and six-month time points after supplement intake. The control group was not given any supplementation but was also followed at the same time points.

### 2.9. Sample Size Determination

Clinically, the efficacy of LM was unknown at the time of this study. A recent report [10] evaluated the changes in serum carotenoids, MPOD and contrast sensitivity in a group of healthy individuals with intake of the LM and PV supplements. The study had a sample size of 15 in each group and found a significant increase in MPOD and serum carotenoids at the six-month time period. Using the means and standard deviation from the study, a total sample size 34 was considered sufficient to provide a power of 80% while setting alpha error at 5%. 

## 3. Statistical Analysis

Due to the differences in health of an individual’s eyes, the right eye and left eye results were evaluated separately and not combined. The datasets were analyzed using a mixed-design analysis of variance (ANOVA) for each outcome measure and a *p* < 0.05 was considered to be significant. A subanalysis of a paired-samples t-test was performed to evaluate the change in the individual parameters from baseline to different treatment time intervals. All comparisons that showed a *p* < 0.05 were reported and, additionally, a Bonferroni correction was performed to the *p*-value to avoid inflation of alpha, due to repeated significance testing. At the request of the journal’s reviewers, a post-hoc analysis of OCT findings was performed and the asymmetry between the right and left eye of the retina in the LM group and the PV group was reported. 

## 4. Results

Figure 1 provides the details about recruitment and randomization of participants included for analysis. At the end of the trial period, a total of 56 participants had complete reliable measurements and were included within the analysis. The mean age of study participants was 68.4 years (SD 5.30, range 54–80 years), consisting of 21 males and 35 females. No adverse events were reported during this study.

Table 1 outlines the mean values and standard deviation (SD) for age, visual acuity, DAR and MPOD measurements in each group. Baseline parameters were similar across all groups. At baseline, DAR scores demonstrated group differences between the treatment groups and control group (Table 1). There were some changes in DAR measurements tested during follow-up visits. On average a 40 s increase in DAR was seen in the LM group at the six-month period compared to the baseline. Whereas the PV group varied by approximately 15 s and the control group showed a 25 s improvement. These changes observed during the supplementation period were not statistically significant across the treatment groups (Table 1) (*p* > 0.05).

The mean MPOD increased in the LM group when compared to the baseline data (see Table 1). The increase in MPOD was asymmetric, with the right eye showing a greater increase compared to the left eye. The right eye showed an MPOD increase of 0.08, whereas the increase in the left eye 0.04 at the six-month visit compared to the baseline data (paired samples t-test *p*-values 0.04 and 0.13, respectively). In the PV group, the MPOD levels showed small fluctuations in the measured MPOD, which remained unchanged in both eyes at a six-month follow up (see Table 1). A Bonferroni-corrected *p*-value of < 0.0125 was considered to be significant when changes in MPOD were compared to the baseline data. Neither the LM group nor the PV group showed a significant change over time with supplementation in all comparisons (*p* > 0.0125). 

The Table 2 shows the changes in the CSF scores with the use of both supplements. The improvements from LM supplementation were observed after three months in the right eye with CS measurements at 12 and 18 CPD (*p* < 0.05) and the LM group reported improvement at all CPD levels in both eyes (*p* < 0.05) after 6 months. For the PV group, differences were found in the left eye at 18 CPD at 3 months. Improvements at 3, 6 and 18 CPD in the right eye and 3, 12 and 18 in the left eye (*p* < 0.05) were noted at 6 months. Overall, LM supplementation showed significantly better improvements in contrast sensitivity in comparison to the PV group and control groups (*p* < 0.001). The improvements seen from LM supplementation showed a linear change over time, with a tendency to show earlier improvements at the three-month period compared to the PV group. 

A Bonferroni-corrected *p*-value of <0.00625 was considered to be significant while comparing the changes within groups at various contrast thresholds during this study. Using this conservative approach, the LM group shows significant improvement in CSF at 6, 12 and 18 CPD for the right eye data and 6 and 18 CPD for the left eye data following six months of treatment. Neither the control group nor the PV group showed changes in contrast sensitivity during the six-month study period after Bonferroni correction. 

There was an asymmetric improvement in vision in both the LM group and the PV group (see Table 2). Post-hoc, at the request of the reviewers, an analysis of the baseline OCT data obtained on both eyes of each participant was conducted by a single observer (PGD). The number of ETDRS sectors that were automatically flagged outside the 95% of the diversified distribution of normal of OCTs reference database were recorded for each eye. In the LM group, 21 of the 25 participants showed some sectors outside the 95% of limits of normality in an OCT macula cube 512 × 128 scan. Of these, 19 participants showed asymmetric macula thickness and damage as flagged by reference database. Greater damage was seen in the left eye compared to right eye of 13 individuals, whereas 6 individuals showed greater damage in the right eye compared to the left. In the PV group, 7 of the 16 participants had an identifiable sector outside the 95% limits of normality, with 5 individuals showing greater damage in the left eye compared to their right and 1 individual showing greater damage in the right eye compared to the left.

## 5. Discussion

To the best of our knowledge, this is the first clinical study that assessed the benefits of the micronized lipid-based carotenoid supplement Lumega-Z (LM) in a group considered at risk of AMD. This study evaluated the visual benefits and MPOD changes observed with LM supplementation and compared it with the PreserVision (PV) supplementation. The PV supplement is extensively researched and is the current clinical standard in treating individuals with risk of AMD [27], whereas there was a gap in knowledge about the use of the LM supplement in individuals at risk of AMD. There are numerous differences in the LM formulation and the PV formulation (see Appendix A). The LM supplement is a medical food that is a liquid formulation and has a difference in the amount and types of macular carotenoids present compared to the PV supplement, which is a soft gel [10]. The PV supplement has 10 mg of lutein and 2 mg of zeaxanthin. However, the LM supplement has 15 mg of lutein, 3 mg of zeaxanthin and, additionally, 10 mg of meso-zeaxanthin [10]. The LM supplement has numerous other micronutrients and is coupled with intake of omega-3 supplementation. The carotenoids have been shown to have visual benefits in healthy and disease states [1,21,22,23,24,25,26,42,43,44,45] and when their supplementation is coupled with omega-3, the bioavailability of carotenoids is enhanced [46,47].

Due to these fundamental differences in the LM and the PV supplements, it was hypothesized that the LM group would show a greater benefit compared to the PV group. This study was meant to simulate a “real-life” and head-to-head comparison of the two commercially available supplements. The LM and PV were used as per the manufacturer’s guidelines and no attempts were made to either match the levels of ingredients or the delivery system.

During the six-month study period, visual performance as measured using contrast sensitivity showed an improvement in both the LM and the PV group. The visual benefits as measured by assessing contrast sensitivity function in the LM group were significantly higher than those reported in the PV group. In the LM group, a linear change in measured contrast sensitivity was shown over time, with notable increases seen at three months and more prominent improvements at the six-month follow-up visit. The PV group also showed a trend toward improvement but was not statistically significant after Bonferroni correction. Compared to the control group, there was a significant change in the measured CSF in the LM, group. 

Numerous studies [25,26,42,43,44,45,46,47,48,49,50,51,52,53,54,55,56,57] have shown that increased carotenoid intake through oral supplementation can lead to an improvement in visual function as measured by contrast sensitivity. This change is both dose and time dependent and it is expected that a time delay exists between initiation of supplementation with carotenoids and the measured benefits in vision function [25,26,42,43,44,45,46,47,48,49,50,51,52,53,54,55,56,57]. This study confirms the dose-related trend shown by prior reports [25,26,42,43,44,45,46,47,48,49,50,51,52,53,54,55,56,57] and the LM supplementation does increase contrast sensitivity in various spatial frequencies to a greater extent than the PV supplement, which had a lower carotenoid dose and other differences in the formulation compared to LM. The CSV-1000E evaluates CSF in each spatial frequency at 8 different steps/gradations. The mean improvement in CSF seen in the LM group compared to the baseline data was approximately 0.11, 0.14, 0.18 and 0.21 log units, which on average represents a one- to two-step improvement in contrast sensitivity. This level of improvement would represent a clinically significant improvement, as it one step higher than the population norms for the age group [33]. 

The carotenoid intake is also cumulative in its effect and even though the PV group did not show a statistically significant improvement in contrast sensitivity function in this trial, there was a trend toward improved CSF. One could expect that with a longer duration of intake of the PV supplement, there would be a proportionate improvement in CSF. It is not possible to determine what differences in the LM supplement were responsible for the improvement in visual function in this study as we did not have individual groups for each nutrient. There are numerous formulation differences in the LM supplement and the PV supplement, with the LM supplement having more micronutrients (see Appendix A). Additionally, the amount of carotenoid, formulation differences and the intake of omega-3 fish oil supplement could have all played a role.

Improvements in CSF measurements were seen in both eyes for the LM group, but a subanalysis revealed varying levels of improvement between the right and left eyes (Table 2). These differences in visual benefits may be associated with retinal pathology and the early effects of macular degeneration. Asymmetry of retinal health between the eyes of individual participants was indeed visible when evaluating the baseline OCT scans. The asymmetry was greater in the LM group compared to the PV group. As indicated by the OCT sectors outside the 95% limits of normality, there was indeed potentially greater damage in the left eyes compared to the right eyes in both the treatment groups, which in part explains the asymmetric and greater visual benefits seen in the right eyes compared to the left eyes. These data suggest that although the carotenoid vitamin supplement was provided orally, the absorption or bioavailability of the supplement is variable between eyes with asymmetric damage. These findings should be interpreted with caution as these were not from a primary analysis and were an outcome of a secondary post-hoc analysis to explain the results.

As expected, the mean values for visual acuity did not show any improvements in any group. The ETDRS chart uses high-contrast black letters on a white background to evaluate the visual acuity threshold. It is designed primarily as a measure of defocus or optical blur and is principally used for prescribing spectacles or contact lenses. The visual acuity in individuals with retinal drusen and at risk of AMD or early non-exudative AMD remains close to 20/20. There is limited potential of improvement and it is not surprising that visual acuity is relatively insensitive to the changes in visual function in individuals with this disease and its treatment. 

There were some changes in DAR measurements in all groups. These changes observed were likely due to measurement-related noise as one expects in clinical measurements. The DAR did not show statistically significant improvements with carotenoid intake over time. The findings agree with a prior report that showed a lack of correlation between MPOD and rod-mediated response and function [58]. The fact that the study participants were individuals with subclinical AMD, and not overt AMD, may have also played a role in this outcome. Although the treatment groups had a statistically significantly longer DAR than the control group, the overall deficiencies in DAR were limited compared to controls (Table 1). As such, the potential for improvement was probably low during the relatively short time period of this study and longer trials and future studies are needed to determine whether supplementation of carotenoids for longer periods will yield to improvement in DAR in individuals at risk of AMD. 

The secondary outcome was to evaluate the changes in MPOD with the supplementation of various carotenoids. Numerous studies have shown that MPOD can be augmented with oral supplementation in healthy individuals [10,21,22,49,50,51,53,54,55] and in individuals with early macular degeneration [23,24,25,42,45,48,52,57,59,60]. In the present study, the MPOD levels did not show a significant change during a six-month study period with either LM or PV supplementation. The discrepancy in this study’s findings compared to the prior reports could stem from differences in the duration of carotenoid supplementation in the studies. Whereas the present study was a six-month supplementation trial, prior studies that showed a significant increase in measured MPOD in early macular degeneration needed a 12 month [25,45,48,57,59,60] or 24 month [57] intake of oral supplements to show a measurable increase in MPOD. MPOD is a useful biomarker and its levels correlate with retinal health [23,24,25,42,45,48,52,57,59,60] and various cognitive functions [61,62,63,64,65,66,67,68]. Further, the levels of carotenoids in brain, especially those found in the occipital lobe [69], correlate with macular carotenoid levels [70]. Future studies are required to determine the duration of LM supplementation needed to elucidate a measurable increase in MPOD in individuals at risk of AMD.

It was previously shown that visual function could improve with carotenoids and an associated MPOD density improvement is visible [25,26,42,43,44,45,46,47,48,49,50,51,52,53,54,55,56,57]. In the present study, although MPOD did not improve significantly, we did see an improvement in CSF in both the treatment groups. Stringham et al. [50] have postulated that mechanisms other than elevated MPOD can be responsible for improvement in CSF. They proposed that lateral inhibition sensitivity with MPOD augmentation was related to improvement in contrast sensitivity. Particularly, the increasing antioxidant capacity possibly leading to enhancement of neurophysiology of the retina by increasing the metabolic efficiency of visual cycle, which in turn can also have advantages for post-receptoral circuitry [50]. In other words, getting a more “redox homeostasis” could lead to a better visual function along with other neural processes [71]. This also has support with numerous studies showing the increased cognitive role of carotenoids [61,62,63,64,65,66,67,68]. 

Comparing the results of the present study to our prior work [10], we find that the CSF improved in the present study, whereas the CSF was not significantly improved in the prior paper [10]. This is not completely surprising as our prior work was a six-month trial [10], with individuals who had visual acuity at peak levels of 20/20 or better. In these healthy individuals with contrast sensitivity function already at optimal levels, the testing will need to be more sensitive and will need a wider range of contrast sensitivity, particularly testing the upper end of visibility, in order to be able to measure improvements. The MPOD levels improved significantly in the previous study [10] but not in the present study. This difference can be explained in part due to two factors: (1) age and (2) level of oxidative damage. The prior study [10] involved healthy young adults who perhaps have better absorption of the LM supplement and lower oxidative demands compared to individuals with drusen and subclinical AMD. The results of the present study would indicate that it is relatively easier to improve CSF in individuals who have decreased vision, but carotenoid vitamin supplementation will be needed for a longer time to obtain a significant measurable MPOD increase. Further research in the factors that determine the elevation of MPOD after supplementation are warranted. 

One of the limitations of the present study was the reliance on self-reported intake of supplements and stabilization of dietary habits. Additional limitations include relatively small sample sizes, a short trial period and the inability to truly mask supplement assignment between the treatment groups (Lumega-Z is a liquid and AREDS-2 is a soft gel). It is indeed possible that longer durations may benefit both the treatment groups. It is also possible that a longer study duration may have shown an improvement in MPOD and dark adaptation recovery time. Another shortcoming of this study was the use of heterochromatic flicker photometry [41]. Although it is commonly used clinically, it is prone to error due to its subjective nature. Heterochromatic flicker photometry due to its small footprint is perhaps most common clinical device utilized to measure MPOD [4,8,10,15,16,26,41,44,45,48,66,67,72,73]. However, objective techniques such as dual-wavelength autofluorescence [74] or macular pigment reflectometry [21,75,76] could decrease variability in results, given that the participants in this study were clinical patients and not trained participants in a psychophysical study. A learning curve may account for the increase in CSF testing overtime in healthy controls. Although the improvement in CSF was not statistically significant in the healthy group, a trend showing improvement was visible. Additionally, macular pigment reflectometry could provide in vivo carotenoid measurements of the lutein and zeaxanthin levels, which would be a welcome addition to the biomarker MPOD [21,75,76].

The present study showed that LM supplementation in individuals at risk of AMD improves CSF. The improvements in CSF were significantly greater and quicker in the LM group than when using the AREDS-2 formulation in the PV group. The exact advantages of the quicker and greater improvements seen with LM supplementation are not known but one could hypothesize that there would be improvements in patient quality of life related to vision, cognitive function and perhaps disease progression, as seen in prior carotenoid supplementation studies, but this will require further research. Longitudinal studies with larger sample sizes are needed to confirm these findings and to fully quantify the benefits of carotenoid uptake in the retina. Additional studies are also needed in order to establish the mechanisms responsible for the visual improvements from carotenoid supplementation. Studies focused on electrophysiological testing and optical coherence tomography (OCT) angiography may shed further light on additional, more subtle, oculophysiological benefits of carotenoid supplementation. 

## 6. Conclusions

This open label RCT on individuals with sub-clinical AMD, showed a greater and more rapid improvement in contrast sensitivity function with supplementation of Lumega-Z when compared to supplementation with PreserVision. The differences in formulation, quantity of macular carotenoids and other nutrients, could explain some of the visual benefits seen with Lumega-Z supplementation.

## Figures and Tables

**Table 1:** Visual function measurements in various groups.

	Lumega-Z Group *n* = 25		PreserVision Group *n* = 16		Control *n* = 15	
Age, years, mean (SD)	68.9 (5.5)		68.4 (4.4)		67.9 (5.5)	
Visual Acuity, mean (SD)	OD	OS	OD	OS	OD	OS
Baseline	25.4 (5.2)	25.9 (5.0)	23.1 (3.8)	25.3 (4.0)	23.3 (3.6)	23.9 (4.5)
3 months	24.6 (6.0)	24.8 (4.5)	23.3 (3.9)	24.3 (3.7)	22.3 (3.7)	24.6 (6.9)
6 months	25.7 (7.3)	24.8 (5.2)	23.8 (3.8)	24.7 (4.0)	22.3 (3.7)	24.3 (5.49)
Dark adaptation recovery (minutes), mean (SD)	OD	OS	OD	OS	OD	OS
Baseline	7.1 (2.5)	6.3 (2.4)	7.1 (1.6)	6.9 (1.7)	5.3 (1.6)	5.2 (1.7)
3 months	7.2 (2.9)	7.1 (2.7)	6.9 (2.5)	6.7 (2.1)	5.3 (1.3)	5.7 (1.6)
6 months	7.6 (3.9)	7.2 (3.2)	6.9 (2.1)	6.9 (1.8)	5.6 (1.5)	5.1 (1.8)
MPOD mean (SD)	OD	OS	OD	OS	OD	OS
Baseline	0.41 (0.21)	0.38 (0.24)	0.42 (0.22)	0.39 (0.22)	0.38 (0.14)	0.41 (0.15)
3 months	0.44 (0.21)	0.41 (0.21)	0.38 (0.22)	0.40 (0.20)	0.43 (0.15)	0.46 (0.12)
6 months	0.49 (0.23)	0.42 (0.23)	0.39 (0.12)	0.38 (0.19)	0.49 (0.12)	0.42 (0.15)

Abbreviations: MPOD, macular pigment optical density; SD, standard deviation.

**Table 2:** Mean contrast sensitivity function measured by CSV-1000E in various groups.

		Baseline				Three Months				Six Months			
		A	B	C	D	A	B	C	D	A	B	C	D
Lumega-Z- Group *n* = 25	OD	1.58	1.79	1.45	0.96	1.59	1.88	**1.57**	**1.11**	**1.71**	1.95 *	1.63 *	1.19 *
		(0.21)	(0.18)	(0.21)	(0.23)	(0.22)	(0.21)	**(0.23)**	**(0.18)**	**(0.12)**	**(0.21)**	**(0.21)**	**(0.31)**
	OS	1.58	1.80	1.49	1.04	1.59	1.86	1.61	1.12	**1.67**	1.94 *	**1.67**	1.24 *
		(0.17)	(0.11)	(0.11)	(0.17)	(0.20)	(0.20)	(0.23)	(0.24)	**(0.14)**	**(0.15)**	**(0.19)**	**(0.16)**
PreserVision Group *n* = 16	OD	1.62	1.88	1.56	1.08	1.73	1.95	1.61	**1.19**	**1.73**	**1.97**	1.64	**1.26**
		(0.20)	(0.21)	(0.23)	(0.25)	(0.15)	(0.19)	(0.23)	**(0.21)**	**(0.16)**	**(0.15)**	(0.21)	**(0.18)**
	OS	1.59	1.85	1.49	1.08	1.63	1.89	1.59	1.19	**1.70**	1.93	**1.60**	**1.26**
		(0.18)	(0.23)	(0.25)	(0.30)	(0.17)	(0.19)	(0.16)	(0.20)	**(0.15)**	(0.19)	**(0.19)**	**(0.20)**
Control *n* =15	OD	1.69	1.88	1.58	1.12	1.71	1.91	1.62	1.20	1.76	1.95	1.66	1.26
		(0.12)	(0.10)	(0.17)	(0.21)	(0.13)	(0.12)	(0.17)	(0.28)	(0.13)	(0.12)	(0.15)	(0.20)
	OS	1.64	1.88	1.58	1.11	1.69	1.96	1.64	1.22	1.74	1.92	1.67	1.20
		(0.22)	(0.13)	(0.15)	(0.12)	(0.14)	(0.12)	(0.15)	(0.19)	(0.22)	(0.18)	(0.15)	(0.18)

Abbreviations: A is 3 cycles per degree (CPD), B is 6 CPD, C is 12 CPD, and D is 18 CPD. Notes: Bolded values are statistically significant values (*p* < 0.05) relative to the other groups using pairwise comparisons. Bold values with a * represent *p* < 0.001.

## Appendix A

**Formula** **Products**	**Guardion Health Sciences Lumega-Z**	**Bausch + Lomb PreserVision AREDS-2 Formula**
Vitamin C	500 mg	500 mg
Thiamin	1.5 mg	
Riboflavin	1.7 mg	
Niacin	20 mg	
Vitamin B6	10 mg	
Folate	800 mcg	
Vitamin B12	1000 mcg	
Vitamin D3	2000 IU	
Vitamin E	200 IU	400 IU
Vitamin K		
Biotin	100 mcg	
Pantothenic Acid	10 mg	
Calcium	250 mg	
Iodine		
Magnesium	100 mg	
Zinc	25 mg	80 mg
Selenium	70 mg	
Copper	3 mg	2 mg
Manganese	2 mg	
Chromium	120 mcg	
Molybdenum	75 mcg	
NAC	500 mg	
POA Blend	200 mg	
Acetyl-L-Carnitine	500 mg	
Taurine	500 mg	
Quercetin	100 mg	
CoQ10	50 mg	
Lycopene	500 mcg	
Lutein	15 mg	10 mg
Zeaxanthin	3 mg	2 mg
Meso-Zeaxanthin	10 mg	
Astaxanthin	4000 mcg	
	Omega Boost	
Fish Oil Concentrate	1260 mg	
DHA (Docosahexaenoic Acid)	675 mg	
EPA (Eicosapentaenoic Acid)	230 mg	
